# High Prevalence of Overweight and Its Association with Mid-Upper Arm Circumference among Female and Male Farmers in Tanzania and Mozambique

**DOI:** 10.3390/ijerph18179128

**Published:** 2021-08-30

**Authors:** Laila Eleraky, Ramula Issa, Sónia Maciel, Hadijah Mbwana, Constance Rybak, Jan Frank, Wolfgang Stuetz

**Affiliations:** 1 Department of Food Biofunctionality, Institute of Nutritional Sciences, University of Hohenheim, 70599 Stuttgart, Germany; laila.eleraky@nutres.de (L.E.); ramulajuma.issa@uni-hohenheim.de (R.I.); jan.frank@nutres.de (J.F.); 2 Faculty of Health Sciences, Lúrio University, Nampula 3100, Mozambique; smaciel@unilurio.ac.mz; 3 Department of Food Technology, Nutrition and Consumer Sciences, Sokoine University of Agriculture, Morogoro 3006, Tanzania; hadija27@yahoo.com; 4 Leibniz Centre for Agricultural Landscape Research (ZALF), 15374 Müncheberg, Germany; constance.rybak@zalf.de

**Keywords:** overweight, body mass index, mid-upper arm circumference, Tanzania, Mozambique, Africa

## Abstract

The increasing prevalence of overweight/obesity may already have reached the farmers in Tanzania and Mozambique. Here, the measurement of the mid-upper-arm-circumference (MUAC) could become a simple and sensitive tool for early detection of at-risk groups of overweight as well as underweight. Body Mass Index (BMI) and MUAC of female and male farmers (*n* = 2106) from different regions of Tanzania and the Zambézia province, Mozambique, were analyzed by region, sex, age, and correlates. MUAC cut-offs, calculated via BMI cut-offs (<18.5, ≥25, and ≥30 kg/m^2^), and multiple linear regression (MLR), compared to those selected by highest Youden’s index (YI) value, were assessed. The study showed an overall higher prevalence of overweight (19%) than underweight (10%) due to the high number of overweight female farmers (up to 35%) in southern Tanzania. BMI, which was mainly and positively predicted by MUAC, was higher in Tanzania and among female farmers, and decreased significantly from the age of ≥65 years. MUAC cut-offs of <24 cm and ≥30.5 cm, calculated by MLR, detected 55% of farmers being underweight and 74% being overweight, with a specificity of 96%; the higher cut-off <25 cm and lower cut-off ≥29 cm, each selected according to YI, consequently detected more underweight (80%) and overweight farmers (91%), but on the basis of a lower specificity (87–88%). Overweight was evident among female farmers in East Africa. MUAC cut-offs, whether defined via linear regression or Youden’s Index, could prove to be easy-to-use tools for large-scale screenings of both underweight and overweight.

## 1. Introduction

As early as the 1990s, the World Health Organization underlined that overweight and obesity are becoming a major health problem in developing countries, especially among adult women [1]. A meta-analysis from 2016 with more than 19.2 million adult women and men from 186 different countries revealed an increase of global age-standardised BMI from 1975 to 2014 in both sexes: the prevalence of obesity (BMI ≥ 30 kg/m^2^) increased from 3.2% to 10.8% in men, and from 6.4% to 14.9% in women; compared with the increase in obesity, the prevalence of underweight decreased (by a smaller amount) from 13.8% to 8.8% in men and from 14.6% to 9.7% in women [2]. Adequate nutritional status is defined as the physiological state of an individual that results from balance between nutrient intake and the specific requirements of the human body [3]. Malnutrition refers to all forms of inadequate nutrition, including undernutrition (BMI < 18.5 kg/m^2^), overweight (BMI ≥ 25 kg/m^2^, pre-obesity and obesity), obesity (BMI ≥ 30 kg/m^2^), and micronutrient malnutrition [4,5]. A nutrition transition from undernutrition towards overweight and obesity in urban and rural areas of Tanzania and Mozambique was attributed to changes in dietary patterns [6,7]. This transition is characterised by a high consumption of food items that are high in fats, refined carbohydrates, and sugars, and a low intake of vegetables and fibres [6]. A high prevalence of overweight has been previously reported among Mozambican adolescent girls living in rural areas [7]. Similarly, the prevalence of pre-obesity (23%) and obesity (17%) was high in females and males from the Kilimanjaro region in Tanzania, where women were more than five times as likely (adjusted OR 5.5 (3.1–9.8)) to be overweight (BMI ≥ 25 kg/m^2^) compared to men [8]. 

The nutritional status is usually assessed by measuring weight and height and the calculation of the body mass index (BMI) [9]. The mid-upper-arm circumference (MUAC), which is the circumference of the left arm midway between the tip of the elbow and the tip of the shoulder, was repeatedly validated as a fast and reliable approach for assessing the nutritional status of a population [10,11,12]. Nevertheless, the MUAC is still not established as an alternative method and indicator of nutritional status and malnutrition beside the BMI. A systematic review of 47 cross-sectional and longitudinal studies revealed low maternal MUAC as a predictor for poor pregnancy outcomes (low birth weight), and showed a strong association of low MUAC (<22, 23, 24 cm) with low BMI (<18.5 kg/m^2^) among adults in low-income countries across Africa and Asia [11]. A meta-analysis compiling 17 datasets from Africa, Asia, and North and South America found a strong positive correlation between MUAC and BMI and that MUAC cut-offs in the range of 23.0 to <25.5 cm could serve as appropriate indicators for low BMI, while a MUAC < 24.0 cm meets the criteria across various subpopulations when assessed against BMI < 18.5 kg/m^2^ [13]. The strong positive correlation between MUAC and BMI and its role as a valuable anthropometric marker and nutritional status indicator for undernutrition was confirmed in studies among adolescent girls in Central Mozambique and adults in Bangladesh [14,15]. A multi-cross-sectional study in Nigeria, South Africa, Uganda, and Tanzania (*n* = 1463) revealed a high incidence of overweight and obesity, which far surpassed and replaced undernutrition as a public health problem in both rural and urban areas [16]; therefore, MUAC and appropriate MUAC cut-offs could be a simple and sensitive tool for early detection of at risk-groups of overweight as well as underweight.

In the present study, we assessed anthropometrics of female and male farmers from the baseline surveys of the Scale-N and the Vegi-Leg projects. Both projects aim to ameliorate the nutritional status of small-scale farmers through tailored nutrition-sensitive interventions. The Scale-N project aimed to achieve food and nutrition security of small-scale farmers in Central Tanzania by the development of nutrition-sensitive and diversified agricultural production methods and improvement of nutritional behavior [17,18,19]; The Vegi-Leg project operates in Lindi region, South Tanzania, and in Gurue, Zambézia province of Mozambique among pigeon pea *(Cajanus cajan)* farmers, in areas with a high prevalence of anaemia and micronutrient deficiencies with the aim of safeguarding perennial nutrition security through the development of low-cost processing technologies for nutrient-dense products from pigeon peas and dark green leafy vegetables [20,21,22].

The objectives of the present study were to analyze and evaluate (1) the nutritional status among female and male farmers in Tanzania and Mozambique using BMI and MUAC, (2) the correlations between BMI with MUAC considering sex, study region, and age, and (3) MUAC cut-offs as appropriate markers to identify undernutrition as well as overnutrition.

## 2. Materials and Methods

### 2.1. Study Population and Design

**Figure 1:**
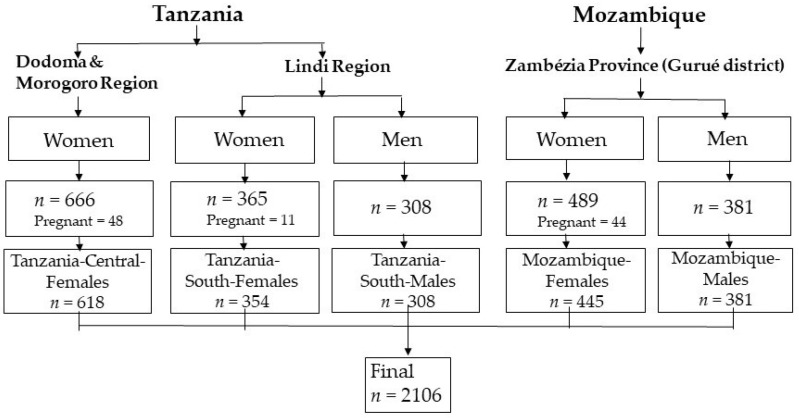
Study population: female and male farmers from Tanzania and Mozambique.

The study population included female farmers of the Scale-N and female and male pigeon pea farmers of the Vegi-Leg projects. A total of 666 female self-sufficient small-scale farmers (mothers and/or caregivers) aged 20 to 75 years, 85% of whom were of reproductive age (15–49 years) and 3.6% ≥65 years, were enrolled in the Scale-N project from July to August 2016 in four different villages in the Dodoma and Morogoro regions, Central Tanzania [17,18,19]. Between July and August 2019, the Vegi-Leg project enrolled 673 farmers from two villages in the Lindi region of Southern Tanzania, aged 16 to 89 years, and 870 farmers from two villages in the Gurué district of Zambézia province in Central Mozambique, aged 18 to 65 years (Figure 1) [20]; of the farmers from Lindi and Gurué, 16% and 3.7% were ≥65 years old, respectively, while 64% and 81% of the female farmers were of reproductive age. 

The surveys were carried out according to the guidelines laid down in the ‘Declaration of Helsinki’ and approved by the National Institute for Medical Research and the Ministry of Health, Community Development, Gender, Elderly and Children in Dar es Salaam (NIMR/HQ/R.8a/Vol. IX/2226) and Dodoma, Tanzania (NIMR/HQ/R.8a/Vol.IX/ 3040), and ethically reviewed by the National Bioethics Committee for Health of Mozambique (IRB00002657, Ref 370/CNBS/19). Written informed consent was obtained from all farmers. After excluding 103 pregnant women, anthropometric data from a total of 2106 female and male farmers from the different provinces in Tanzania and Mozambique were included in the present investigation.

### 2.2. Anthropometric Measurements

Weight, height, and mid-upper arm circumference (MUAC) were measured using electronic or mechanical floor scales (Seca 874 in Tanzania, Seca 750 in Mozambique, Seca GmbH & Co KG Hamburg, Germany), a wooden (UNICEF) or plastic stadiometer (Seca 213, Seca GmbH & Co KG Hamburg, Germany), and a standard MUAC tape (UNICEF in Tanzania or Seca 201 in Mozambique), respectively. Weight was recorded to the nearest 0.1 kg, while height and MUAC were measured to the nearest 0.1 cm. MUAC was measured on the left arm. Body mass index (BMI) was calculated from weight and height measured at admission; standard cut-offs for underweight (<18.5 kg/m^2^), overweight (≥25 kg/m^2^), obesity (≥30 kg/m^2^), and low MUAC for adults (<24 cm) were used according to WHO and FANTA (Food and Nutrition Technical Assistance) instructions [4,19]. The term overweight (or crude overweight) is used to define a BMI ≥ 25 kg/m^2^, while pre-obesity refers to a BMI range from ≥25 kg/m^2^ to <30 kg/m^2^ [23].

### 2.3. Statistical Analyses 

Age and anthropometrics (weight, height, BMI) were described using medians and interquartile ranges (IQR: 25% and 75% percentile) for continuous variables and frequencies (%) for categorical data. Differences of age and anthropometrics between study regions by sex were compared using Kruskal–Wallis and post-hoc Dunn–Bonferroni test or Chi Square tests (prevalence), as appropriate. 

The association of BMI with MUAC for all farmers and by region and sex was shown via scatter plots and corresponding coefficients of determination (R^2^), while individual relationships of BMI with MUAC, weight, height, age, and elders (>65 years) were presented and evaluated using Spearman’s rank correlation coefficients. Multiple linear regression analysis (with a forward stepwise approach) was applied to identify independent and significant predictors of BMI. The resulting formula (BMI = 0.974 × MUAC + 0.834 × TZ + 0.374 × female + 0.552 × age ≥65 y − 5.447) with MUAC as the main predictor was applied to calculate MUAC cut-offs for all studied farmers, and by country, sex, and the elderly (≥65 years) using the established BMI cut-offs (<18.5, ≥25.0, and ≥30.0 kg/m^2^) for underweight, overweight and obesity [23,24]. Matches (percent of joint true positive cases) between MUAC and BMI categories as well as the sensitivity (true positive/ (true positive + false negative)) and specificity (true negative/(true negative + false positive) of the calculated MUAC cut-offs in identifying BMI categories were compared with matches, sensitivity and specificity of MUAC cut-offs (and respective categories) selected according to the highest Youden’s Index values (YI = sensitivity + specificity − 1). All statistical analyses were conducted using SPSS version 20; two-tailed *p*-values < 0.05 were considered statistically significant.

## 3. Results

A total of 2106 non-pregnant female and male farmers were assessed on anthropometrics (Table 1). The participants consisted of 618 women from the Scale-N baseline study in central Tanzania and 1488 female and male farmers from the Vegi-Leg project in Lindi region, Tanzania, and Zambézia province, Mozambique. The total study population had a median age of 39 years and consisted of 67% female farmers. Farmers from Lindi region, South Tanzania, had the highest median age, and participating men in Lindi region and Zambézia province were older than the corresponding women. 

The median weight and height of all farmers was 52.8 kg and 155.6 cm; male farmers were heavier (53.9 vs. 52.2 kg) and significantly taller (162 vs. 153.4 cm), but had a significantly lower BMI and MUAC than female farmers (Table 1). In terms of BMI categories, 18.8% were overweight, including 4.6% with obesity, compared to 10.3% of all farmers who were underweight. In both countries, there was a higher prevalence of pre-obesity and obesity, and thus a significantly higher prevalence of overweight among female compared to male farmers. Further, overweight in female farmers was higher in Tanzania than in Mozambique, with the highest prevalence among female farmers of the Lindi province in South Tanzania (35% with BMI > 25 kg/m^2^). One in four (26%) and one in three (35%) female farmers in central and southern Tanzania, respectively, was overweight. In contrary, 15% of the female Mozambican farmers and less than 9% of all male farmers were overweight. 

The overall median MUAC was 27.2 cm, ranging from 26.4 cm for male farmers from Mozambique to 28.1 cm for female farmers from Lindi; accordingly, male farmers had a significantly higher prevalence of low MUAC (<24 cm) compared to female farmers, in Tanzania as well as in Mozambique. The 80th and 95th percentiles of MUAC were 30 and 33.5 cm, respectively; these cut-offs showed a significantly higher prevalence of high MUAC among female versus male farmers, particularly evident in Lindi, Tanzania. 

**Figure 2:**
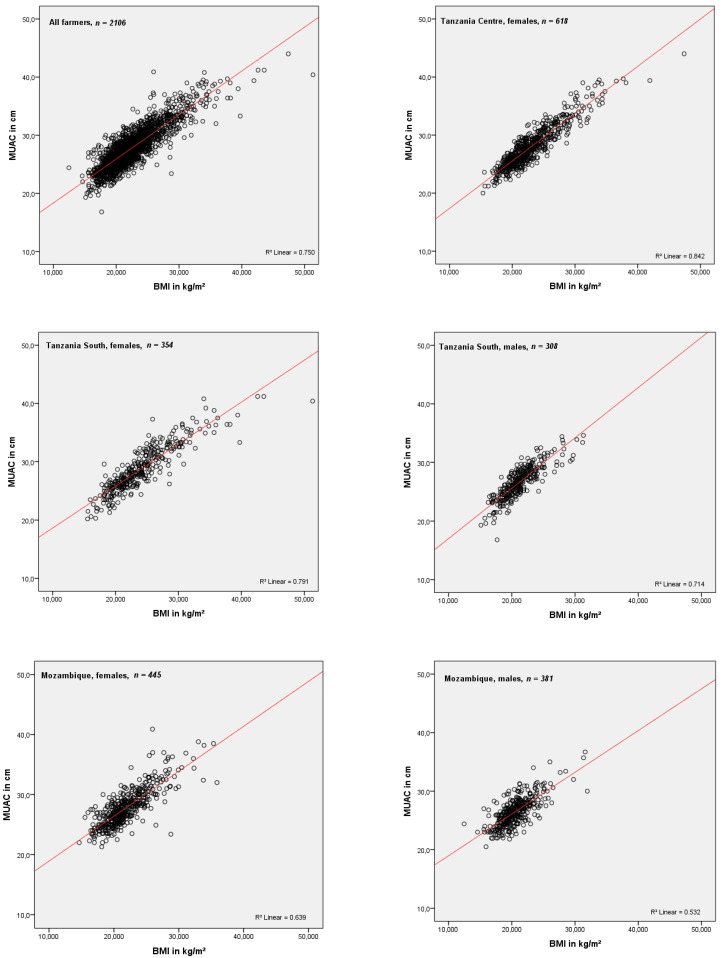
Association of mid-upper arm circumference (MUAC) with body mass index (BMI) in farmers and the respective sub-groups by sex and region.

Linear regression analysis revealed strong positive correlations of BMI with MUAC within the entire study population as well as the individual subgroups in terms of region and sex (Figure 2). The coefficient of determination (R^2^) was higher in Tanzanian than in Mozambican farmers and overall higher among female farmers in both countries than for corresponding male farmers. Accordingly, the highest coefficients of determination were shown for the female farmers of Tanzania (R^2^ = 0.842 and 0.791), who also represented the groups with the highest prevalence of overweight; in contrary, the lowest coefficient was shown for male farmers from Mozambique (R^2^ = 0.532), who had the lowest prevalence of overweight. 

The correlations of BMI with MUAC, weight, height, age, and age ≥ 65 years among all farmers and clustered by sex and region are shown in Table 2: In the entire study population and both countries, the BMI correlated strongly and positively with MUAC (r = 0.837, 0.885, and 0.758). The correlations were stronger in Tanzania than in Mozambique and in female than in male farmers. BMI was inversely correlated with height, age and the elderly (≥65 years); the inverse correlation of BMI with age and elderly was strongest in male farmers from Tanzania.

Multiple linear regression analysis revealed MUAC as a decisive and main predictor of BMI (Table 3). The MUAC was highly positively associated with BMI (partial r = 0.862). Further country (Tanzania vs. Mozambique), sex, and the elderly (age ≥65 years) were significantly associated with BMI in the final ‘adjusted’ model.

Table 4 show the assessments of calculated and selected MUAC cut-offs for underweight, overweight, and obesity by multiple linear regression and highest Youden’s Index (YI). It also shows the slightly higher proportion of underweight among men and the elderly and again underlines the high proportion of Tanzanian women who are overweight and obese. MUAC cut-offs for malnutrition calculated by linear regression and BMI values (<18.5, ≥25, ≥30 kg/m^2^) ranged from 23.2 to 24.6 cm for underweight, from 29.8 to 31.2 cm for overweight, and from 35.0 to 36.4 cm for obesity in the individual subgroups, and were finally for ‘all farmers’ rounded to <24 cm, ≥30.5 cm, and ≥35.5 cm. These MUAC cut-offs identified 55.5%, 73.7%, and 53.1% of the farmers as underweight, overweight, and obese, respectively. MUAC cut-offs selected on the basis on the highest Youden’s Index (highest sensitivity/selectivity ratio) were <25 cm, ≥29 cm, and ≥31.5 cm. The higher cut-off for undernutrition (<25 cm) and lower cut-off values for overweight (≥29 cm) and obesity (≥31.5 cm) consequently revealed higher matches of 79.8%, 91.1%, and 95.8%, respectively, while the specificity values of the selected Youden’s Index cut-offs were simultaneously lower compared to those by linear regression: 87% vs. 96% for underweight, 88% vs. 96% for overweight, and 92% vs. 99% for obesity, with the consequence of more false positives and ultimately slightly overestimated numbers of matched or identified prevalence of underweight, overweight, and obesity.

## 4. Discussion

The present study includes three cross-sectional studies and study sites and, therefore, allowed for the examination of significant associations of the BMI and its categories with MUAC in 2106 female and male small-scale farmers from different regions in Tanzania and Mozambique. The results revealed a higher prevalence of overweight (BMI ≥ 25 kg/m^2^) than of underweight (BMI < 18.5 kg/m^2^) and low MUAC (<24 cm). Female farmers from Tanzania were particularly affected by overweight and obesity, which pose serious public health concerns (26% and 35% with BMI ≥ 25 kg/m^2^) according to WHO classification [24]. 

The results confirm the increasing prevalence of overweight among African women of reproductive age, as recently reported in context with metabolic risk factors (e.g., anaemia, hypertension) from 33 sub-Saharan countries, including Tanzania and Mozambique [25]. The records of over 13,000 women from Tanzania (in 2015) and over 13,500 women from Mozambique (in 2011) showed a high prevalence of overweight (pre-obesity plus obesity) with 28% and 17%, respectively [21,26]. In Tanzania, the prevalence of undernutrition in women remained unchanged between the demographic health surveys of 2004–2005 and 2015–2016, whereas the prevalence of overweight (pre-obesity and obesity) rose from 18% to 28%; this high figure is in line with the female farmers of the present study living in southern and central Tanzania, where one in three and one in four women respectively were overweight. The increasing rates of overweight (BMI ≥ 25 kg/m^2^) and obesity (BMI ≥ 30 kg/m^2^) are very likely related to food consumption patterns that have changed from consuming fibre-rich foods to refined foods and overall higher amounts of sugar and fats; these include high amounts of refined maize flour (‘Ugali’), strongly sweetened teas, deep fried pastry products, oil and stir-fried foods, which are now easily affordable and available even in rural areas [8,27]. The dominance of cereal-based diets with insufficient fruit and vegetable consumption, despite high local food biodiversity of plant- based products, has been frequently observed in rural areas of Tanzania and Mozambique [18,28]. Surveys in African countries in the 1990s have already shown that the burden of overweight among adult women (20–49 years) was higher than that of underweight in both urban and rural areas [29]. In 1996, 28% of Tanzanian women from urban areas compared to 11% living in rural settings were overweight, while there was no significant difference in the prevalence of underweight (8.6% in urban vs. 9.6% in rural areas); the prevalence of overweight in rural areas (11%) has now doubled and tripled in the female Tanzanian farmers of the present study. Likewise, female and male adults in rural and peri-urban East Uganda (*n* = 1210) had an overall higher prevalence of pre-obesity (18% with BMI 25–29.99 kg/m^2^) than of underweight (7%), but only because of the very high prevalence (twice that of men) of pre-obese women [30]. Similarly, a study of 435 female and male adults (between 18 and 45 years) from rural and urban communities of Nigeria revealed that the prevalence of pre-obesity (22%) was much higher and of obesity (4%) was still higher than the prevalence of underweight (2%); 40% of the urban and 30% of all rural females (twice as many women as men in both urban and rural areas) were either pre-obese or obese, and 43% of the urban and 31% of the rural females had either an increased (waist circumference ≥ 80 cm) or substantially increased risk (waist circumference ≥ 88 cm) for metabolic syndrome [31]. A study among 976 adults (606 females and 370 males) in two regional capital cities of Ghana, showed that over half of the participants from Takoradi (54%) compared to one third (38%) from Cape Coast were either pre-obese or obese; prevalence of pre-obesity and in particular of obesity was several fold higher in women than in men, and the study population had an overall high prevalence of hypertension (27.0%), diabetes (34.0%), and mild to severe anaemia (47%) [32].

The present study revealed that the MUAC as an anthropometric marker correlates strongly and positively with the BMI and its defined categories and therefore offers very good prerequisites for the detection of underweight, overweight and obesity. Our MUAC cut-offs for underweight of <24 cm calculated via multiple linear regression and of <25 cm selected via highest Youden’s index identified 55% and 78%, respectively, of the farmers with underweight. MUAC cut-offs in the range of ≤23.5 to ≤25.0 cm and a proposed global MUAC cut-off of <24 cm for underweight in adults cm were recently suggested in a multicentre study combining twenty compiled datasets from different countries, including seven from Africa [12]. Although the higher cut-off at <25 cm selected by Youden’s Index identified more underweight farmers, the specificity decreased from 96% to 87%, meaning the number of false positives increased and the true agreement on prevalence of underweight, compared to the lower cut-off of <24 cm calculated via linear regression, was slightly overestimated. Both MUAC cut-offs, <24 and <25 cm, identified more Tanzanian, male and the elderly (≥65 years) farmers with underweight, but again with a consequently lower specificity. A recent study in 302 female and male chronically ill and healthy adults from two urban public hospitals in Nepal suggested a MUAC cut-off of 24.5 cm (according to highest Youden’s Index) for both sexes to identify underweight [33]. 

The present study is one of the first to evaluate specific MUAC cut-offs for the screening of overnutrition, i.e., overweight and obesity. The MUAC cut-off of ≥30.5 cm calculated via linear regression and of ≥29 cm selected via highest Youden’s index were able to detect 74% and 90% of farmers, respectively, with ‘general’ overweight (pre-obesity plus obesity). Due to the lower ‘Youden’s-Index-selected’ cut-off, one consequently gets more farmers and matches with overweight farmers, but simultaneously the specificity decreased from 96% to 88% and consequently, the number of false positives (farmers classified as overweight while having a BMI in the normal range) increased, and the true prevalence of overweight is slightly overestimated. More older people were reached via the MUAC cut-offs for underweight, whereas fewer older people were now reached via the MUAC cut-offs for overweight, suggesting more studies with a bigger sample size in order to assess specific MUAC-cut-offs for the elderly (≥65 years). In addition, the MUAC appears to be a more appropriate marker than BMI for assessing poor nutritional status for older people. A study among older female and male Dutch participants (*n* = 1307 ≥ 65 years) revealed a reduction of MUAC within a three years period, irrespective of changes in body weight, which was explained by muscular atrophy and bone loss in older age [34]. Furthermore, a study from Malawi confirms our findings that more attention to under- not overnutrition should be paid among the elderly [35]. In general, MUAC was judged more appropriate than BMI for detecting underweight in older adults with kyphosis, in individuals with changes in body composition, and in the acute phase of emergencies, e.g., adults with oedema due to protein-energy malnutrition, where thus weight gain is not related to nutritional status [36,37].

Lower cut-offs for overweight of 27.7 and 27.9 cm were also identified for male and female adolescents (15–19 years) living in Addis Ababa, Ethiopia [38]; this suggests that MUAC could be influenced by factors such as age, and thus appropriate cut-offs for specific age groups such as adolescents and the elderly should be evaluated. Furthermore, MUAC itself can be considered a valuable additional and independent marker for assessing nutritional status, rather than just a simpler method and substitute of the BMI.

In regard to obesity, the 95th percentile of our study population was 33.5 cm, very close to a proposed and by linear regression (and BMI of 30 kg/m^2^) calculated ‘obesity’ MUAC cut-off for adults in South Africa of >33.0 cm [39]; this cut-off of 33 cm, derived from data of the national South African demographic health survey, is to our knowledge the only suggested MUAC cut-off for obesity so far. In this survey, ‘African’ women had a more than three times higher prevalence of obesity (32% vs. 6%) and high MUAC (31% vs. 10% > 33 cm) than African men. Our calculated MUAC cut-off of ≥35.5 cm via linear regression identified 53% and the much lower cut-off of ≥31.5 cm, selected via highest Youden’s index, accordingly detected as many as 96% of obese farmers. These cut-offs were significantly different from each other with consequently different levels of agreements but also very different grades of specificities; a lower cut-off (as already shown with the MUAC cut-off for overweight) logically reaches more people but with a lower specificity, while the higher cut-off reaches fewer people, but the severe cases of obesity with a very high specificity. However, the evaluation of obesity in our study was mainly related to women, especially in southern Tanzania. Therefore, larger studies in East Africa and other African countries could confirm our results and would also allow to assess specific MUAC cut-offs of underweight, overweight and obesity by different ethnic, sex, and age groups.

The strength of the present study is the large sample size and the assessment of both women and men, who all belong to a homogenous study population, namely, small-scale farmers from different rural areas of Tanzania and Mozambique. For these farmers, we had a complete set of data on BMI and MUAC; however, one limitation of the study is that subgroups such as the elderly and those with obesity are still underrepresented for the assessment of respective specific cut-offs; in particular, for obesity as a high-risk factor for metabolic syndrome and adverse health outcomes an even larger sample for reliable cut-offs are required. A further limitation is the cross-sectional design with the associated single measurements as well as the different teams for each country and thus the probability of deviations in consistent measurement quality.

## 5. Conclusions

The outcomes of the present study confirm a growing and, according to the high numbers, serious health problem of over-nutrition among female small-scale farmers in Tanzania, while at the same time a relatively large number of male farmers in Tanzania and Mozambique still suffer from underweight. This is one of the first studies suggesting MUAC cut-offs to identify overweight and obesity in Africa. MUAC could be a reliable tool for the screening of both under- and overnutrition, thus making it a promising valuable anthropometric and nutritional marker in field studies, in particular where scales and stadiometers are not accessible or impractical for large-scale studies.

## Figures and Tables

**Table 1:** Characteristics and anthropometrics of the farmers during baseline studies.

	All (*n* = 2106)	Tanzania-Centre-Females (*n* = 618)	Tanzania-South-Females (*n* = 354)	Tanzania-South-Males (*n* = 308)	Mozambique-Females (*n* = 445)	Mozambique- Males (*n* = 381)
Age [years] ^1^	39.0 (29.0, 49.0)	36.0 ^a^ (30.0, 44.0)	42.0 ^b^ (32.0, 58.0)	44.0 ^b^ (32.0, 58.7)	35.0 ^c^ (25.0, 45.0)	38.0 ^d^ (27.0, 51.0)
Age ≥ 65 years ^2^	7.5 (159)	3.6 (22)	15.8 (56)	16.2 (50)	2.9 (13)	4.7 (18)
Weight [kg] ^1^	52.8 (47.9, 59.6)	53.3 ^a^ (48.1, 60.5)	53.6 ^a,b^ (47.6, 62.2)	55.0 ^b^ (49.3, 61.2)	50.2 ^c^ (45.9, 56.0)	53.0 ^a^ (49.9, 59.0)
Height [cm] ^1^	155.6 (151.1, 161.0)	154.5 ^a^ (151.0, 158.1)	151.7 ^b^ (148.2, 155.5)	162.1^c^ (158.0, 166.7)	153.1 ^d^ (148.9, 156.8)	162.0 ^c^ (157.1, 166.6)
BMI [kg/m^2^] ^1^	21.5 (19.7, 24.0)	22.2 ^a^ (20.4, 25.0)	23.4 ^b^ (20.9, 26.3)	21.0 ^c^ (19.5, 22.7)	21.3 ^d^ (19.8, 23.7)	20.3 ^e^ (19.1, 21.7)
BMI categories ^2^						
<18.5 kg/m^2^	10.3 (216)	6.3 (39)	6.8 (24)	13.0 (40)	11.2 (50)	16.5 (63)
18.5–24.9 kg/m^2^	71.0 (1495)	68.1 (421)	58.5 (207)	78.6 (242)	73.0 (325)	78.7 (300)
25–29.9 kg/m^2^	14.2 (299)	19.1 (118)	23.4 (83)	7.5 (23)	13.5 (60)	3.9 (15)
≥30 kg/m^2^	4.6 (96)	6.5 (40)	11.3 (40)	1.0 (3)	2.2 (10)	0.8 (3)
BMI ≥ 25 kg/m^2^	18.8 (395)	25.6 (158) ^a^	34.7 (123) ^b^	8.5 (26) ^c^	15.7 (70) ^d^	4.7 (18) ^e^
MUAC cut-offs ^2^						
MUAC < 24 cm	9.4 (199)	7.4 (46) ^a^	7.6 (27) ^a^	14.9 (46) ^b^	6.1 (27) ^a^	13.9 (53) ^b^
MUAC ≥ 30 cm	20.7 (435)	24.1 (149) ^a^	33.6 (119) ^b^	10.1 (31) ^c^	23.1 (103) ^a^	8.7 (33) ^c^
MUAC ≥ 33.5 cm	5.4 (114)	7.4 (46) ^a,c^	11.0 (39) ^a^	1.3 (4) ^b^	4.7 (21) ^c^	1.0 (4) ^b^

Data are median (25th and 75th percentile) ^1^, or percentage (number of farmers) ^2^, all such values. Differences between individual groups were assessed by Kruskall–Wallis and post-hoc Dunn–Bonferroni tests (pairwise comparisons) or Chi Square tests; values within a row not sharing a common superscript letter ^(a,b,c,d,e)^ are significantly different at *p* < 0.05. Chi Square tests (pravelences) within BMI categories: *p* < 0.001. BMI ≥ 25 kg/m^2^: overweight; MUAC = mid-upper arm circumference; BMI = body mass index; MUAC ≥ 30 cm and ≥ 33.5 cm representing 80th and 95th percentile, respectively.

**Table 2:** Correlation of BMI with MUAC, weight, height, age, and age ≥ 65 among all farmers, and clustered by sex and country.

BMI [kg/m^2^]	MUAC [cm]	Weight [kg]	Height [cm]	Age [years]	Age ≥ 65 y
All, *n* = 2106	0.837 **	0.800 **	−0.154 **	−0.068	−0.098 **
All-Females, *n* = 1417	0.858 **	0.886 **	−0.034	−0.026	−0.053 *
All-Males, *n* = 689	0.774 **	0.791 **	0.038	−0.102 **	−0.154 **
Tanzania, *n* = 1280	0.885 **	0.833 **	−0.135 **	−0.145 **	−0.161 **
TZ-Females, *n* = 972	0.895 **	0.890 **	−0.055	−0.080 *	−0.100 **
TZ-Males, *n* = 308	0.852 **	0.832 **	0.096	−0.255 **	−0.259 **
Mozambique, *n* = 826	0.758 **	0.731 **	−0.150 *	−0.014	−0.027
MZ-Females, *n* = 445	0.786 **	0.867 **	−0.016	0.034	0.042
MZ-Males, *n* = 381	0.703 **	0.743 **	−0.016	−0.035	−0.079

Spearman correlations, * *p* < 0.05; ** *p* < 0.001; TZ= Tanzania; MZ= Mozambique.

**Table 3:** Multiple linear regression analyses assessing predictors of BMI [kg/m^2^].

	Beta	95% CI	*R* Zero-Order	*R* Partial	*p* Value
(constant)	−5.447	−6.117–−4.777			
MUAC, cm	0.974	0.950–0.999	0.866	0.862	<0.001
Country, TZ = 1	0.834	0.667–1.001	0.209	0.209	<0.001
Sex, female = 1	0.374	0.199–0.550	0.260	0.091	<0.001
Age ≥ 65y (=1)	0.552	0.249–0.856	−0.083	0.078	<0.001

Multiple linear regression analysis (with a forward approach): MUAC [cm], region (TZ (Tanzania) = 1 vs. MZ =0), sex (female = 1 vs. male = 0), age ≥ 65years (*n* = 159; =1 vs. age < 65 = 0), and age [years] were included as independent variables in the initial model. BMI = 0.974 × MUAC + 0.834 × TZ +0.374 × female + 0.552 × age ≥ 65y − 5.447; *n* = 2106, R^2^ = 0.768.

**Table 4:** Identification of farmers with underweight, overweight, and obesity by MUAC cut-offs calculated with multiple linear regression vs. those selected by highest Youden’s Index.

				MLR				Y. I.		
BMI Category	% (*n*) BMI Category	MUAC by MLR	% (*n*) BMI Category	Sensiti-Vity [%]	Specifi-City [%]	Y.I.	% (*n*) BMI Category	Sensiti-Vity [%]	Specifi-City [%]	Highest Y. I.
**Underweight**	**<18.5 kg/m^2^**	**24 cm**	**<24 cm**	**<24 cm**	**<24 cm**	**<24 cm**	**<25 cm**	**<25 cm**	**<25 cm**	**<25 cm**
All, *n* = 2106	10.3 (216)	23.7	55.5 (120)	54.6	95.7	0.55	79.8 (168)	77.8	87.2	0.67
Tanzania, *n* = 1280	8.0 (103)	23.3	68.9 (71)	68.0	95.8	0.64	91.3 (94)	91.3	87.2	0.78
Mozambique, *n* = 826	13.7 (113)	24.2	43.4 (49)	42.5	95.5	0.38	65.5 (74)	65.5	87.2	0.53
Female, *n* = 1417	8.0 (113)	23.3	50.4 (57)	50.4	96.7	0.47	77.9 (88)	77.9	89.1	0.67
Male, *n* = 689	14.9 (103)	24.6	61.2 (63)	59.2	93.5	0.53	77.7 (80)	77.7	82.9	0.61
Age ≥ 65y, *n* = 159	22.0 (35)	23.2	80.0 (28)	77.1	85.5	0.63	91.4 (32)	91.4	75.0	0.66
**Overweight**	**≥25 kg/m^2^**	**30.5 cm**	**≥30.5 cm**	**≥30.5 cm**	**≥30.5 cm**	**≥30.5 cm**	**≥29 cm**	**≥29 cm**	**≥29 cm**	**≥29 cm**
All, *n* = 2106	18.8 (395)	30.4	73.7 (291)	72.5	96.2	0.69	91.1 (360)	90.4	87.8	0.78
Tanzania, *n* = 1280	24.0 (307)	30.0	74.3 (228)	73.4	96.5	0.70	91.8 (282)	91.2	87.0	0.78
Mozambique, *n* = 826	10.7 (88)	30.9	71.6 (63)	69.3	95.8	0.65	88.6 (78)	88.6	87.1	0.76
Female, *n* = 1417	24.8 (351)	30.0	75.2 (264)	74.4	95.5	0.70	91.7 (322)	91.2	85.5	0.77
Male, *n* = 689	6.4 (44)	31.2	61.4 (27)	56.8	97.4	0.54	86.4 (38)	86.4	89.6	0.76
Age ≥ 65y, *n* = 159	14.5 (23)	29.8	52.2 (12)	52.2	98.5	0.51	73.9 (17)	73.9	94.1	0.68
**Obesity**	**≥30 kg/m^2^**	**35.5 cm**	**≥35.5 cm**	**≥35.5 cm**	**≥35.5 cm**	**≥35.5 cm**	**≥31.5 cm**	**≥31.5 cm**	**≥31.5 cm**	**≥31.5 cm**
All, *n* = 2106	4.6 (96)	35.5	53.1 (51)	51.0	99.5	0.50	95.8 (92)	95.8	92.1	0.88
Tanzania, *n* = 1280	6.5 (83)	35.1	53.0 (44)	50.6	99.7	0.50	97.6 (81)	97.6	90.7	0.88
Mozambique, *n*= 826	1.6 (13)	36.0	53.8 (7)	53.8	99.1	0.53	84.6 (11)	84.6	94.5	0.79
Female, *n* = 1417	6.4 (90)	35.1	54.4 (49)	52.2	99.2	0.51	96.6 (87)	96.7	89.2	0.86
Male, *n* = 689	0.9 (6)	36.4	33.3 (2)	33.3	1	0.33	83.3 (5)	83.3	97.8	0.81
Age ≥ 65y, *n* = 159	3.8 (6)	35.0	66.7 (4)	50.0	1	0.50	83.3 (5)	83.3	97.4	0.81

Data are % (number), MUAC in cm, and sensitivity and specificity in %; MLR = multiple linear regression, Y.I. = Youden’s Index; MUAC equation (*n* = 2106): MUAC [cm] = (BMI [kg/m^2^] − 0.834 (if TZ) − 0.374 (if female) − 0.552 (if age ≥ 65 years) + 5.447)/0.974.

## Data Availability

Not applicable.
